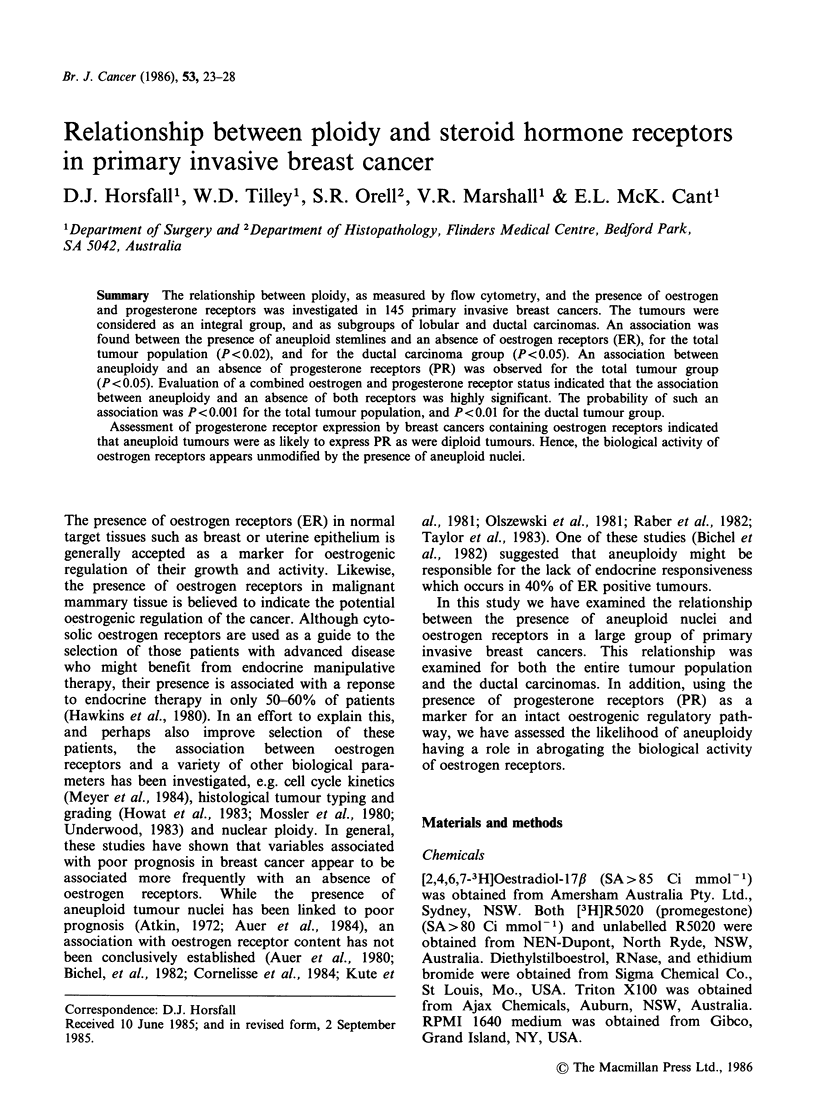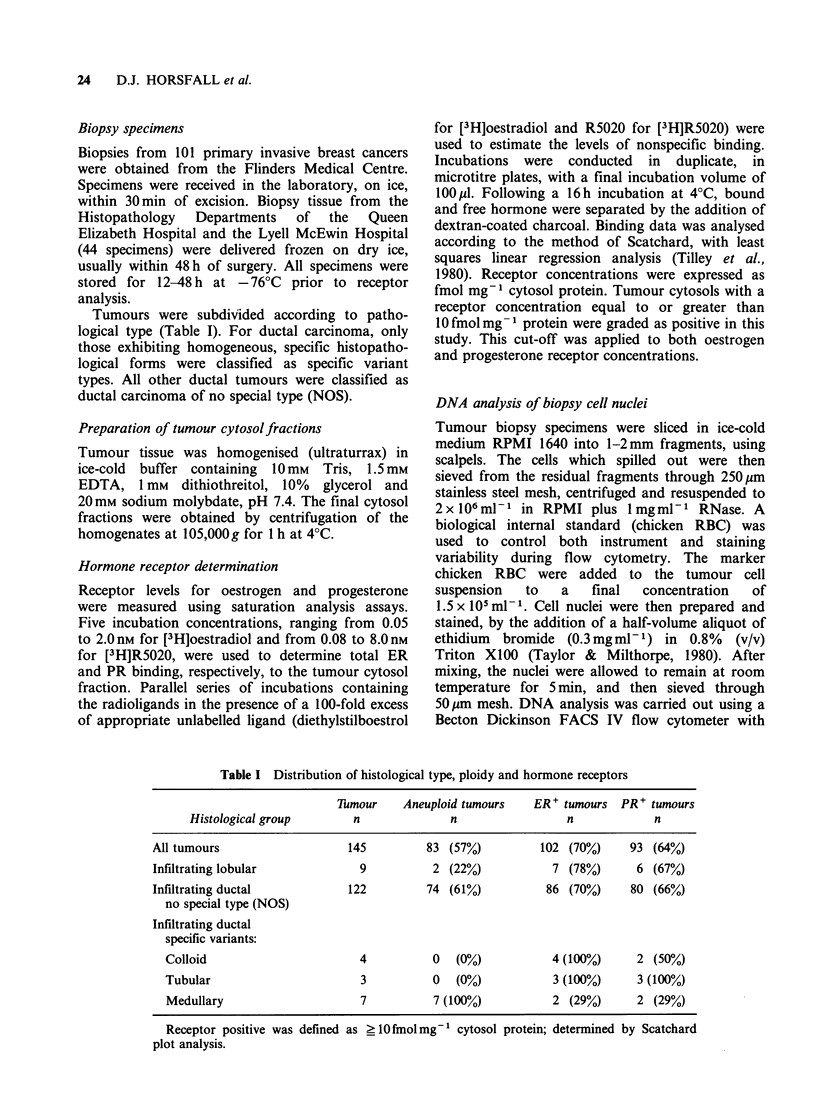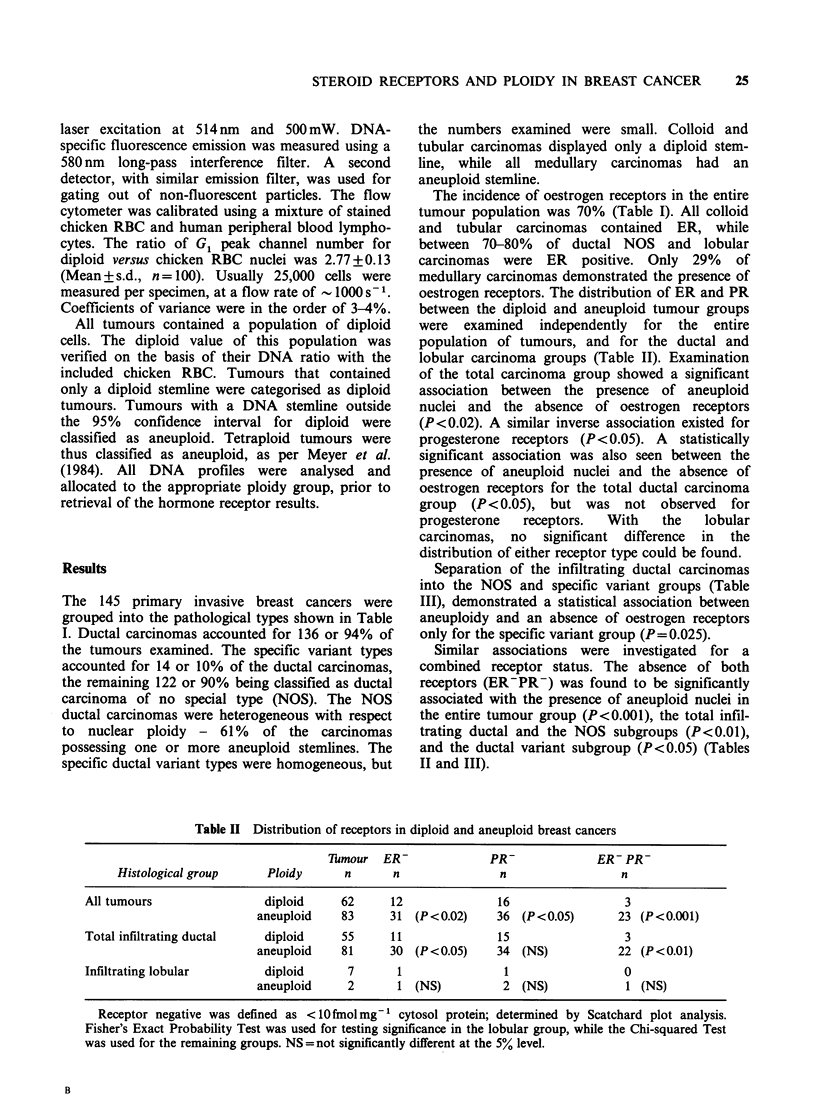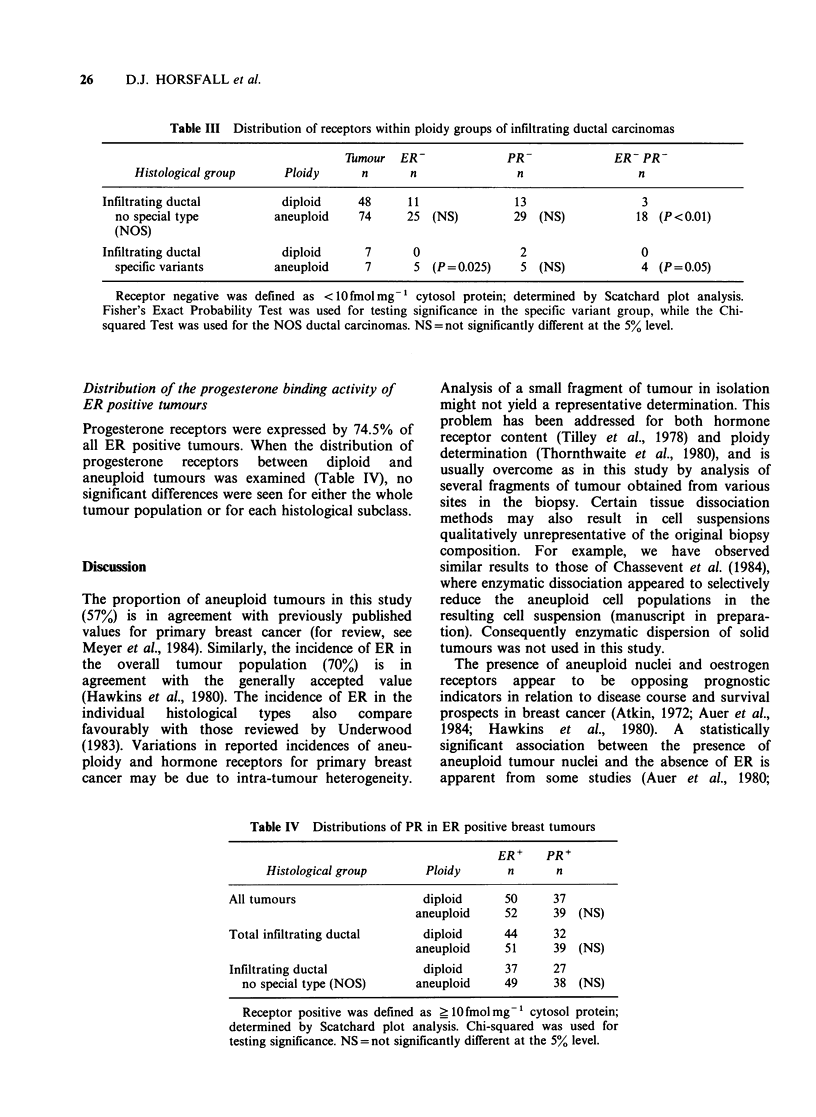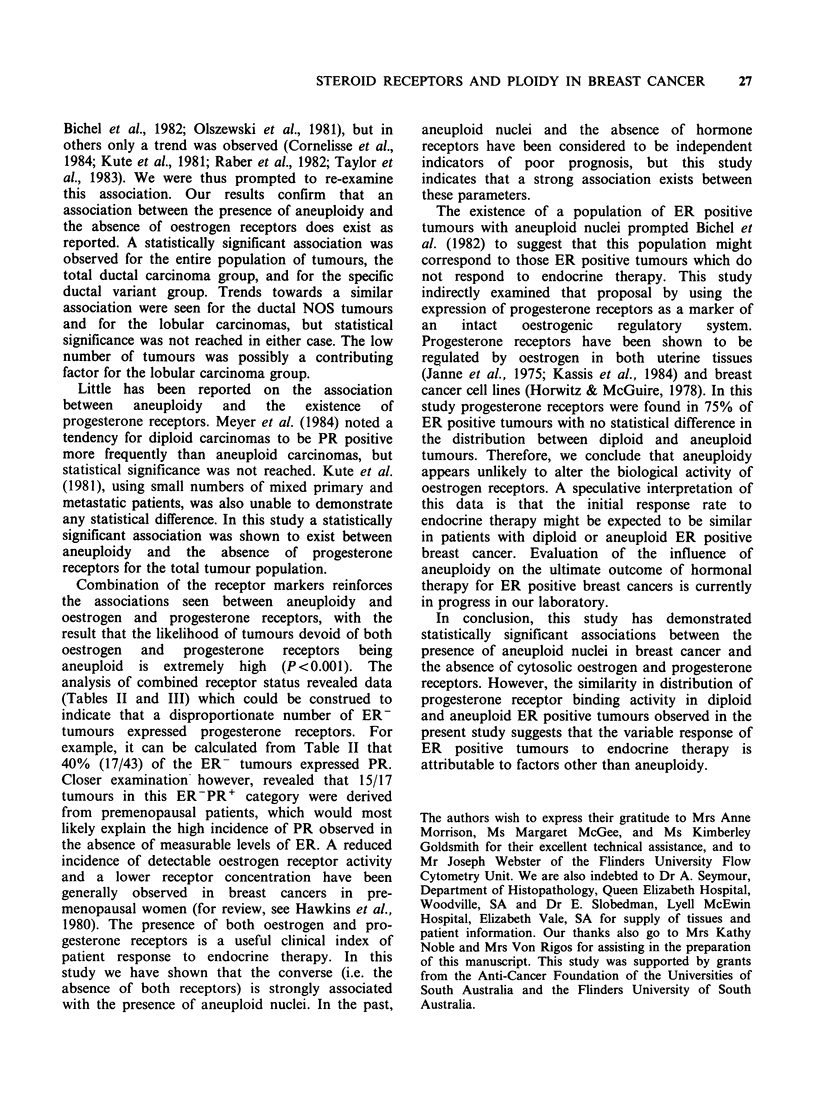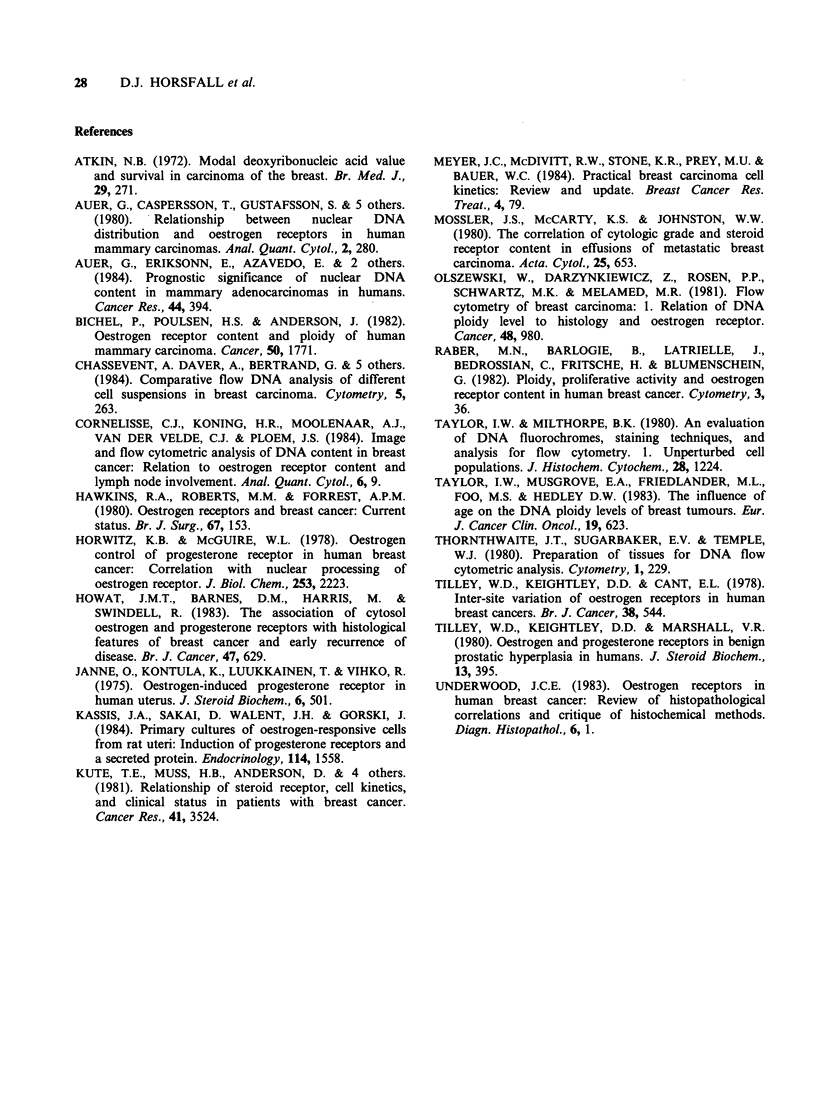# Relationship between ploidy and steroid hormone receptors in primary invasive breast cancer.

**DOI:** 10.1038/bjc.1986.4

**Published:** 1986-01

**Authors:** D. J. Horsfall, W. D. Tilley, S. R. Orell, V. R. Marshall, E. L. Cant

## Abstract

The relationship between ploidy, as measured by flow cytometry, and the presence of oestrogen and progesterone receptors was investigated in 145 primary invasive breast cancers. The tumours were considered as an integral group, and as subgroups of lobular and ductal carcinomas. An association was found between the presence of aneuploid stemlines and an absence of oestrogen receptors (ER), for the total tumour population (P less than 0.02), and for the ductal carcinoma group (P less than 0.05). An association between aneuploidy and an absence of progesterone receptors (PR) was observed for the total tumour group (P less than 0.05). Evaluation of a combined oestrogen and progesterone receptor status indicated that the association between aneuploidy and an absence of both receptors was highly significant. The probability of such an association was P less than 0.001 for the total tumour population, and P less than 0.01 for the ductal tumour group. Assessment of progesterone receptor expression by breast cancers containing oestrogen receptors indicated that aneuploid tumours were as likely to express PR as were diploid tumours. Hence, the biological activity of oestrogen receptors appears unmodified by the presence of aneuploid nuclei.


*Br. J. Cancer* (1986), **53**, 23–28

### Relationship between ploidy and steroid hormone receptors in primary invasive breast cancer

D.J. Horsfall[1], W.D. Tilley[1], S.R. Orell[2], V.R. Marshall[1] & E.L. McK. Cant[1]

[1]*Department of Surgery and* [2]*Department of Histopathology, Flinders Medical Centre, Bedford Park, SA 5042, Australia*

**Summary** The relationship between ploidy, as measured by flow cytometry, and the presence of oestrogen and progesterone receptors was investigated in 145 primary invasive breast cancers. The tumours were considered as an integral group, and as subgroups of lobular and ductal carcinomas. An association was found between the presence of aneuploid stemlines and an absence of oestrogen receptors (ER), for the total tumour population ($P<0.02$), and for the ductal carcinoma group ($P<0.05$). An association between aneuploidy and an absence of progesterone receptors (PR) was observed for the total tumour group ($P<0.05$). Evaluation of a combined oestrogen and progesterone receptor status indicated that the association between aneuploidy and an absence of both receptors was highly significant. The probability of such an association was $P<0.001$ for the total tumour population, and $P<0.01$ for the ductal tumour group.

Assessment of progesterone receptor expression by breast cancers containing oestrogen receptors indicated that aneuploid tumours were as likely to express PR as were diploid tumours. Hence, the biological activity of oestrogen receptors appears unmodified by the presence of aneuploid nuclei.

The presence of oestrogen receptors (ER) in normal target tissues such as breast or uterine epithelium is generally accepted as a marker for oestrogenic regulation of their growth and activity. Likewise, the presence of oestrogen receptors in malignant mammary tissue is believed to indicate the potential oestrogenic regulation of the cancer. Although cytosolic oestrogen receptors are used as a guide to the selection of those patients with advanced disease who might benefit from endocrine manipulative therapy, their presence is associated with a reponse to endocrine therapy in only 50–60% of patients (Hawkins *et al.*, 1980). In an effort to explain this, and perhaps also improve selection of these patients, the association between oestrogen receptors and a variety of other biological parameters has been investigated, e.g. cell cycle kinetics (Meyer *et al.*, 1984), histological tumour typing and grading (Howat *et al.*, 1983; Mossler *et al.*, 1980; Underwood, 1983) and nuclear ploidy. In general, these studies have shown that variables associated with poor prognosis in breast cancer appear to be associated more frequently with an absence of oestrogen receptors. While the presence of aneuploid tumour nuclei has been linked to poor prognosis (Atkin, 1972; Auer *et al.*, 1984), an association with oestrogen receptor content has not been conclusively established (Auer *et al.*, 1980; Bichel, *et al.*, 1982; Cornelisse *et al.*, 1984; Kute *et al.*, 1981; Olszewski *et al.*, 1981; Raber *et al.*, 1982; Taylor *et al.*, 1983). One of these studies (Bichel *et al.*, 1982) suggested that aneuploidy might be responsible for the lack of endocrine responsiveness which occurs in 40% of ER positive tumours.

In this study we have examined the relationship between the presence of aneuploid nuclei and oestrogen receptors in a large group of primary invasive breast cancers. This relationship was examined for both the entire tumour population and the ductal carcinomas. In addition, using the presence of progesterone receptors (PR) as a marker for an intact oestrogenic regulatory pathway, we have assessed the likelihood of aneuploidy having a role in abrogating the biological activity of oestrogen receptors.

### Materials and methods

### *Chemicals*

[2,4,6,7-$^3$H]Oestradiol-17$\beta$ (SA>85 Ci mmol$^{-1}$) was obtained from Amersham Australia Pty. Ltd., Sydney, NSW. Both [$^3$H]R5020 (promegestone) (SA>80 Ci mmol$^{-1}$) and unlabelled R5020 were obtained from NEN-Dupont, North Ryde, NSW, Australia. Diethylstilboestrol, RNase, and ethidium bromide were obtained from Sigma Chemical Co., St Louis, Mo., USA. Triton X100 was obtained from Ajax Chemicals, Auburn, NSW, Australia. RPMI 1640 medium was obtained from Gibco, Grand Island, NY, USA.

Correspondence: D.J. Horsfall
Received 10 June 1985; and in revised form, 2 September 1985.

© The Macmillan Press Ltd., 1986

*Biopsy specimens*

Biopsies from 101 primary invasive breast cancers were obtained from the Flinders Medical Centre. Specimens were received in the laboratory, on ice, within 30 min of excision. Biopsy tissue from the Histopathology Departments of the Queen Elizabeth Hospital and the Lyell McEwin Hospital (44 specimens) were delivered frozen on dry ice, usually within 48 h of surgery. All specimens were stored for 12–48 h at −76°C prior to receptor analysis.

Tumours were subdivided according to pathological type (Table I). For ductal carcinoma, only those exhibiting homogeneous, specific histopathological forms were classified as specific variant types. All other ductal tumours were classified as ductal carcinoma of no special type (NOS).

*Preparation of tumour cytosol fractions*

Tumour tissue was homogenised (ultraturrax) in ice-cold buffer containing 10 mM Tris, 1.5 mM EDTA, 1 mM dithiothreitol, 10% glycerol and 20 mM sodium molybdate, pH 7.4. The final cytosol fractions were obtained by centrifugation of the homogenates at 105,000 *g* for 1 h at 4°C.

*Hormone receptor determination*

Receptor levels for oestrogen and progesterone were measured using saturation analysis assays. Five incubation concentrations, ranging from 0.05 to 2.0 nM for [$^3$H]oestradiol and from 0.08 to 8.0 nM for [$^3$H]R5020, were used to determine total ER and PR binding, respectively, to the tumour cytosol fraction. Parallel series of incubations containing the radioligands in the presence of a 100-fold excess of appropriate unlabelled ligand (diethylstilboestrol for [$^3$H]oestradiol and R5020 for [$^3$H]R5020) were used to estimate the levels of nonspecific binding. Incubations were conducted in duplicate, in microtitre plates, with a final incubation volume of 100 $\mu$l. Following a 16 h incubation at 4°C, bound and free hormone were separated by the addition of dextran-coated charcoal. Binding data was analysed according to the method of Scatchard, with least squares linear regression analysis (Tilley *et al.*, 1980). Receptor concentrations were expressed as fmol mg$^{-1}$ cytosol protein. Tumour cytosols with a receptor concentration equal to or greater than 10 fmol mg$^{-1}$ protein were graded as positive in this study. This cut-off was applied to both oestrogen and progesterone receptor concentrations.

*DNA analysis of biopsy cell nuclei*

Tumour biopsy specimens were sliced in ice-cold medium RPMI 1640 into 1–2 mm fragments, using scalpels. The cells which spilled out were then sieved from the residual fragments through 250 $\mu$m stainless steel mesh, centrifuged and resuspended to $2 \times 10^6$ ml$^{-1}$ in RPMI plus 1 mg ml$^{-1}$ RNase. A biological internal standard (chicken RBC) was used to control both instrument and staining variability during flow cytometry. The marker chicken RBC were added to the tumour cell suspension to a final concentration of $1.5 \times 10^5$ ml$^{-1}$. Cell nuclei were then prepared and stained, by the addition of a half-volume aliquot of ethidium bromide (0.3 mg ml$^{-1}$) in 0.8% (v/v) Triton X100 (Taylor & Milthorpe, 1980). After mixing, the nuclei were allowed to remain at room temperature for 5 min, and then sieved through 50 $\mu$m mesh. DNA analysis was carried out using a Becton Dickinson FACS IV flow cytometer with

**Table I** Distribution of histological type, ploidy and hormone receptors

| *Histological group* | *Tumour n* | *Aneuploid tumours n* | *ER$^+$ tumours n* | *PR$^+$ tumours n* |
|---|---|---|---|---|
| All tumours | 145 | 83 (57%) | 102 (70%) | 93 (64%) |
| Infiltrating lobular | 9 | 2 (22%) | 7 (78%) | 6 (67%) |
| Infiltrating ductal no special type (NOS) | 122 | 74 (61%) | 86 (70%) | 80 (66%) |
| Infiltrating ductal specific variants: | | | | |
| Colloid | 4 | 0 (0%) | 4 (100%) | 2 (50%) |
| Tubular | 3 | 0 (0%) | 3 (100%) | 3 (100%) |
| Medullary | 7 | 7 (100%) | 2 (29%) | 2 (29%) |

Receptor positive was defined as ≧10 fmol mg$^{-1}$ cytosol protein; determined by Scatchard plot analysis.

laser excitation at 514 nm and 500 mW. DNA-specific fluorescence emission was measured using a 580 nm long-pass interference filter. A second detector, with similar emission filter, was used for gating out of non-fluorescent particles. The flow cytometer was calibrated using a mixture of stained chicken RBC and human peripheral blood lymphocytes. The ratio of $G_1$ peak channel number for diploid *versus* chicken RBC nuclei was $2.77 \pm 0.13$ (Mean $\pm$ s.d., $n=100$). Usually 25,000 cells were measured per specimen, at a flow rate of $\sim 1000\,s^{-1}$. Coefficients of variance were in the order of 3–4%.

All tumours contained a population of diploid cells. The diploid value of this population was verified on the basis of their DNA ratio with the included chicken RBC. Tumours that contained only a diploid stemline were categorised as diploid tumours. Tumours with a DNA stemline outside the 95% confidence interval for diploid were classified as aneuploid. Tetraploid tumours were thus classified as aneuploid, as per Meyer *et al.* (1984). All DNA profiles were analysed and allocated to the appropriate ploidy group, prior to retrieval of the hormone receptor results.

### Results

The 145 primary invasive breast cancers were grouped into the pathological types shown in Table I. Ductal carcinomas accounted for 136 or 94% of the tumours examined. The specific variant types accounted for 14 or 10% of the ductal carcinomas, the remaining 122 or 90% being classified as ductal carcinoma of no special type (NOS). The NOS ductal carcinomas were heterogeneous with respect to nuclear ploidy – 61% of the carcinomas possessing one or more aneuploid stemlines. The specific ductal variant types were homogeneous, but the numbers examined were small. Colloid and tubular carcinomas displayed only a diploid stemline, while all medullary carcinomas had an aneuploid stemline.

The incidence of oestrogen receptors in the entire tumour population was 70% (Table I). All colloid and tubular carcinomas contained ER, while between 70–80% of ductal NOS and lobular carcinomas were ER positive. Only 29% of medullary carcinomas demonstrated the presence of oestrogen receptors. The distribution of ER and PR between the diploid and aneuploid tumour groups were examined independently for the entire population of tumours, and for the ductal and lobular carcinoma groups (Table II). Examination of the total carcinoma group showed a significant association between the presence of aneuploid nuclei and the absence of oestrogen receptors ($P<0.02$). A similar inverse association existed for progesterone receptors ($P<0.05$). A statistically significant association was also seen between the presence of aneuploid nuclei and the absence of oestrogen receptors for the total ductal carcinoma group ($P<0.05$), but was not observed for progesterone receptors. With the lobular carcinomas, no significant difference in the distribution of either receptor type could be found.

Separation of the infiltrating ductal carcinomas into the NOS and specific variant groups (Table III), demonstrated a statistical association between aneuploidy and an absence of oestrogen receptors only for the specific variant group ($P=0.025$).

Similar associations were investigated for a combined receptor status. The absence of both receptors ($ER^-PR^-$) was found to be significantly associated with the presence of aneuploid nuclei in the entire tumour group ($P<0.001$), the total infiltrating ductal and the NOS subgroups ($P<0.01$), and the ductal variant subgroup ($P<0.05$) (Tables II and III).

**Table II** Distribution of receptors in diploid and aneuploid breast cancers

| *Histological group* | *Ploidy* | *Tumour n* | *$ER^-$ n* | | *$PR^-$ n* | | *$ER^-PR^-$ n* | |
|---|---|---|---|---|---|---|---|---|
| All tumours | diploid | 62 | 12 | | 16 | | 3 | |
| | aneuploid | 83 | 31 | ($P<0.02$) | 36 | ($P<0.05$) | 23 | ($P<0.001$) |
| Total infiltrating ductal | diploid | 55 | 11 | | 15 | | 3 | |
| | aneuploid | 81 | 30 | ($P<0.05$) | 34 | (NS) | 22 | ($P<0.01$) |
| Infiltrating lobular | diploid | 7 | 1 | | 1 | | 0 | |
| | aneuploid | 2 | 1 | (NS) | 2 | (NS) | 1 | (NS) |

Receptor negative was defined as $<10\,fmol\,mg^{-1}$ cytosol protein; determined by Scatchard plot analysis. Fisher's Exact Probability Test was used for testing significance in the lobular group, while the Chi-squared Test was used for the remaining groups. NS = not significantly different at the 5% level.

**Table III** Distribution of receptors within ploidy groups of infiltrating ductal carcinomas

| *Histological group* | *Ploidy* | *Tumour n* | *ER$^-$ n* | | *PR$^-$ n* | | *ER$^-$ PR$^-$ n* | |
|---|---|---|---|---|---|---|---|---|
| Infiltrating ductal | diploid | 48 | 11 | | 13 | | 3 | |
| no special type | aneuploid | 74 | 25 | (NS) | 29 | (NS) | 18 | ($P<0.01$) |
| (NOS) | | | | | | | | |
| Infiltrating ductal | diploid | 7 | 0 | | 2 | | 0 | |
| specific variants | aneuploid | 7 | 5 | ($P=0.025$) | 5 | (NS) | 4 | ($P=0.05$) |

Receptor negative was defined as $<10\,\text{fmol}\,\text{mg}^{-1}$ cytosol protein; determined by Scatchard plot analysis. Fisher's Exact Probability Test was used for testing significance in the specific variant group, while the Chi-squared Test was used for the NOS ductal carcinomas. NS = not significantly different at the 5% level.

*Distribution of the progesterone binding activity of ER positive tumours*

Progesterone receptors were expressed by 74.5% of all ER positive tumours. When the distribution of progesterone receptors between diploid and aneuploid tumours was examined (Table IV), no significant differences were seen for either the whole tumour population or for each histological subclass.

### Discussion

The proportion of aneuploid tumours in this study (57%) is in agreement with previously published values for primary breast cancer (for review, see Meyer *et al.*, 1984). Similarly, the incidence of ER in the overall tumour population (70%) is in agreement with the generally accepted value (Hawkins *et al.*, 1980). The incidence of ER in the individual histological types also compare favourably with those reviewed by Underwood (1983). Variations in reported incidences of aneuploidy and hormone receptors for primary breast cancer may be due to intra-tumour heterogeneity. Analysis of a small fragment of tumour in isolation might not yield a representative determination. This problem has been addressed for both hormone receptor content (Tilley *et al.*, 1978) and ploidy determination (Thornthwaite *et al.*, 1980), and is usually overcome as in this study by analysis of several fragments of tumour obtained from various sites in the biopsy. Certain tissue dissociation methods may also result in cell suspensions qualitatively unrepresentative of the original biopsy composition. For example, we have observed similar results to those of Chassevent *et al.* (1984), where enzymatic dissociation appeared to selectively reduce the aneuploid cell populations in the resulting cell suspension (manuscript in preparation). Consequently enzymatic dispersion of solid tumours was not used in this study.

The presence of aneuploid nuclei and oestrogen receptors appear to be opposing prognostic indicators in relation to disease course and survival prospects in breast cancer (Atkin, 1972; Auer *et al.*, 1984; Hawkins *et al.*, 1980). A statistically significant association between the presence of aneuploid tumour nuclei and the absence of ER is apparent from some studies (Auer *et al.*, 1980;

**Table IV** Distributions of PR in ER positive breast tumours

| *Histological group* | *Ploidy* | *ER$^+$ n* | *PR$^+$ n* | |
|---|---|---|---|---|
| All tumours | diploid | 50 | 37 | |
| | aneuploid | 52 | 39 | (NS) |
| Total infiltrating ductal | diploid | 44 | 32 | |
| | aneuploid | 51 | 39 | (NS) |
| Infiltrating ductal | diploid | 37 | 27 | |
| no special type (NOS) | aneuploid | 49 | 38 | (NS) |

Receptor positive was defined as $\geq 10\,\text{fmol}\,\text{mg}^{-1}$ cytosol protein; determined by Scatchard plot analysis. Chi-squared was used for testing significance. NS = not significantly different at the 5% level.

Bichel *et al.*, 1982; Olszewski *et al.*, 1981), but in others only a trend was observed (Cornelisse *et al.*, 1984; Kute *et al.*, 1981; Raber *et al.*, 1982; Taylor *et al.*, 1983). We were thus prompted to re-examine this association. Our results confirm that an association between the presence of aneuploidy and the absence of oestrogen receptors does exist as reported. A statistically significant association was observed for the entire population of tumours, the total ductal carcinoma group, and for the specific ductal variant group. Trends towards a similar association were seen for the ductal NOS tumours and for the lobular carcinomas, but statistical significance was not reached in either case. The low number of tumours was possibly a contributing factor for the lobular carcinoma group.

Little has been reported on the association between aneuploidy and the existence of progesterone receptors. Meyer *et al.* (1984) noted a tendency for diploid carcinomas to be PR positive more frequently than aneuploid carcinomas, but statistical significance was not reached. Kute *et al.* (1981), using small numbers of mixed primary and metastatic patients, was also unable to demonstrate any statistical difference. In this study a statistically significant association was shown to exist between aneuploidy and the absence of progesterone receptors for the total tumour population.

Combination of the receptor markers reinforces the associations seen between aneuploidy and oestrogen and progesterone receptors, with the result that the likelihood of tumours devoid of both oestrogen and progesterone receptors being aneuploid is extremely high ($P<0.001$). The analysis of combined receptor status revealed data (Tables II and III) which could be construed to indicate that a disproportionate number of $ER^-$ tumours expressed progesterone receptors. For example, it can be calculated from Table II that 40% (17/43) of the $ER^-$ tumours expressed PR. Closer examination however, revealed that 15/17 tumours in this $ER^-PR^+$ category were derived from premenopausal patients, which would most likely explain the high incidence of PR observed in the absence of measurable levels of ER. A reduced incidence of detectable oestrogen receptor activity and a lower receptor concentration have been generally observed in breast cancers in pre-menopausal women (for review, see Hawkins *et al.*, 1980). The presence of both oestrogen and progesterone receptors is a useful clinical index of patient response to endocrine therapy. In this study we have shown that the converse (i.e. the absence of both receptors) is strongly associated with the presence of aneuploid nuclei. In the past, aneuploid nuclei and the absence of hormone receptors have been considered to be independent indicators of poor prognosis, but this study indicates that a strong association exists between these parameters.

The existence of a population of ER positive tumours with aneuploid nuclei prompted Bichel *et al.* (1982) to suggest that this population might correspond to those ER positive tumours which do not respond to endocrine therapy. This study indirectly examined that proposal by using the expression of progesterone receptors as a marker of an intact oestrogenic regulatory system. Progesterone receptors have been shown to be regulated by oestrogen in both uterine tissues (Janne *et al.*, 1975; Kassis *et al.*, 1984) and breast cancer cell lines (Horwitz & McGuire, 1978). In this study progesterone receptors were found in 75% of ER positive tumours with no statistical difference in the distribution between diploid and aneuploid tumours. Therefore, we conclude that aneuploidy appears unlikely to alter the biological activity of oestrogen receptors. A speculative interpretation of this data is that the initial response rate to endocrine therapy might be expected to be similar in patients with diploid or aneuploid ER positive breast cancer. Evaluation of the influence of aneuploidy on the ultimate outcome of hormonal therapy for ER positive breast cancers is currently in progress in our laboratory.

In conclusion, this study has demonstrated statistically significant associations between the presence of aneuploid nuclei in breast cancer and the absence of cytosolic oestrogen and progesterone receptors. However, the similarity in distribution of progesterone receptor binding activity in diploid and aneuploid ER positive tumours observed in the present study suggests that the variable response of ER positive tumours to endocrine therapy is attributable to factors other than aneuploidy.

The authors wish to express their gratitude to Mrs Anne Morrison, Ms Margaret McGee, and Ms Kimberley Goldsmith for their excellent technical assistance, and to Mr Joseph Webster of the Flinders University Flow Cytometry Unit. We are also indebted to Dr A. Seymour, Department of Histopathology, Queen Elizabeth Hospital, Woodville, SA and Dr E. Slobedman, Lyell McEwin Hospital, Elizabeth Vale, SA for supply of tissues and patient information. Our thanks also go to Mrs Kathy Noble and Mrs Von Rigos for assisting in the preparation of this manuscript. This study was supported by grants from the Anti-Cancer Foundation of the Universities of South Australia and the Flinders University of South Australia.

### References

ATKIN, N.B. (1972). Modal deoxyribonucleic acid value and survival in carcinoma of the breast. *Br. Med. J.*, **29**, 271.

AUER, G., CASPERSSON, T., GUSTAFSSON, S. & 5 others. (1980). Relationship between nuclear DNA distribution and oestrogen receptors in human mammary carcinomas. *Anal. Quant. Cytol.*, **2**, 280.

AUER, G., ERIKSONN, E., AZAVEDO, E. & 2 others. (1984). Prognostic significance of nuclear DNA content in mammary adenocarcinomas in humans. *Cancer Res.*, **44**, 394.

BICHEL, P., POULSEN, H.S. & ANDERSON, J. (1982). Oestrogen receptor content and ploidy of human mammary carcinoma. *Cancer*, **50**, 1771.

CHASSEVENT, A. DAVER, A., BERTRAND, G. & 5 others. (1984). Comparative flow DNA analysis of different cell suspensions in breast carcinoma. *Cytometry*, **5**, 263.

CORNELISSE, C.J., KONING, H.R., MOOLENAAR, A.J., VAN DER VELDE, C.J. & PLOEM, J.S. (1984). Image and flow cytometric analysis of DNA content in breast cancer: Relation to oestrogen receptor content and lymph node involvement. *Anal. Quant. Cytol.*, **6**, 9.

HAWKINS, R.A., ROBERTS, M.M. & FORREST, A.P.M. (1980). Oestrogen receptors and breast cancer: Current status. *Br. J. Surg.*, **67**, 153.

HORWITZ, K.B. & McGUIRE, W.L. (1978). Oestrogen control of progesterone receptor in human breast cancer: Correlation with nuclear processing of oestrogen receptor. *J. Biol. Chem.*, **253**, 2223.

HOWAT, J.M.T., BARNES, D.M., HARRIS, M. & SWINDELL, R. (1983). The association of cytosol oestrogen and progesterone receptors with histological features of breast cancer and early recurrence of disease. *Br. J. Cancer*, **47**, 629.

JANNE, O., KONTULA, K., LUUKKAINEN, T. & VIHKO, R. (1975). Oestrogen-induced progesterone receptor in human uterus. *J. Steroid Biochem.*, **6**, 501.

KASSIS, J.A., SAKAI, D. WALENT, J.H. & GORSKI, J. (1984). Primary cultures of oestrogen-responsive cells from rat uteri: Induction of progesterone receptors and a secreted protein. *Endocrinology*, **114**, 1558.

KUTE, T.E., MUSS, H.B., ANDERSON, D. & 4 others. (1981). Relationship of steroid receptor, cell kinetics, and clinical status in patients with breast cancer. *Cancer Res.*, **41**, 3524.

MEYER, J.C., McDIVITT, R.W., STONE, K.R., PREY, M.U. & BAUER, W.C. (1984). Practical breast carcinoma cell kinetics: Review and update. *Breast Cancer Res. Treat.*, **4**, 79.

MOSSLER, J.S., McCARTY, K.S. & JOHNSTON, W.W. (1980). The correlation of cytologic grade and steroid receptor content in effusions of metastatic breast carcinoma. *Acta. Cytol.*, **25**, 653.

OLSZEWSKI, W., DARZYNKIEWICZ, Z., ROSEN, P.P., SCHWARTZ, M.K. & MELAMED, M.R. (1981). Flow cytometry of breast carcinoma: 1. Relation of DNA ploidy level to histology and oestrogen receptor. *Cancer*, **48**, 980.

RABER, M.N., BARLOGIE, B., LATRIELLE, J., BEDROSSIAN, C., FRITSCHE, H. & BLUMENSCHEIN, G. (1982). Ploidy, proliferative activity and oestrogen receptor content in human breast cancer. *Cytometry*, **3**, 36.

TAYLOR, I.W. & MILTHORPE, B.K. (1980). An evaluation of DNA fluorochromes, staining techniques, and analysis for flow cytometry. 1. Unperturbed cell populations. *J. Histochem. Cytochem.*, **28**, 1224.

TAYLOR, I.W., MUSGROVE, E.A., FRIEDLANDER, M.L., FOO, M.S. & HEDLEY D.W. (1983). The influence of age on the DNA ploidy levels of breast tumours. *Eur. J. Cancer Clin. Oncol.*, **19**, 623.

THORNTHWAITE, J.T., SUGARBAKER, E.V. & TEMPLE, W.J. (1980). Preparation of tissues for DNA flow cytometric analysis. *Cytometry*, **1**, 229.

TILLEY, W.D., KEIGHTLEY, D.D. & CANT, E.L. (1978). Inter-site variation of oestrogen receptors in human breast cancers. *Br. J. Cancer*, **38**, 544.

TILLEY, W.D., KEIGHTLEY, D.D. & MARSHALL, V.R. (1980). Oestrogen and progesterone receptors in benign prostatic hyperplasia in humans. *J. Steroid Biochem.*, **13**, 395.

UNDERWOOD, J.C.E. (1983). Oestrogen receptors in human breast cancer: Review of histopathological correlations and critique of histochemical methods. *Diagn. Histopathol.*, **6**, 1.